# The widespread adoption of large language model-assisted writing across society

**DOI:** 10.1016/j.patter.2025.101366

**Published:** 2025-10-02

**Authors:** Weixin Liang, Yaohui Zhang, Mihai Codreanu, Jiayu Wang, Hancheng Cao, James Zou

**Affiliations:** 1 Department of Computer Science, Stanford University, Stanford, CA 94305, USA; 2 Department of Electrical Engineering, Stanford University, Stanford, CA 94305, USA; 3 Department of Economics, Stanford University, Stanford, CA 94305, USA; 4 Paul G. Allen School of Computer Science & Engineering, University of Washington, Seattle, WA 98195, USA; 5 Department of Biomedical Data Science, Stanford University, Stanford, CA 94305, USA; 6 Goizueta Business School, Emory University, Atlanta, GA 30322, USA

**Keywords:** large language model, writing

## Abstract

This paper systematically analyzes the adoption of large language models (LLMs), such as ChatGPT, across consumer complaints, corporate press releases, job postings, and United Nations (UN) press releases, covering extensive datasets from January 2022 to September 2024. By late 2024, roughly 18% of financial consumer complaints, 24% of corporate press releases, nearly 10% of job postings in small firms, and 14% of UN press releases involve LLM-assisted writing. Adoption surged rapidly post-ChatGPT release but stabilized by 2024, highlighting generative artificial intelligence (AI)’s broad societal impact and its widespread use across sectors.

## Highlights

• Systematic analysis of 687,241 consumer complaints and 537,413 corporate press releases
• Rapid initial adoption post-ChatGPT, with 24% LLM use in corporate communications by 2024
• 304.3 million job postings show nearly 10% LLM adoption, higher in young firms
• UN press releases (15,919 analyzed) exhibit 14% adoption of generative AI

## The bigger picture

Large language models (LLMs), such as ChatGPT, represent a transformative development in artificial intelligence (AI), significantly impacting how individuals, businesses, and institutions communicate. Yet, despite widespread interest and extensive media coverage, systematic insights into how widely and why different organizations adopt these powerful tools remain limited. Understanding adoption patterns across various sectors—including consumer interactions, corporate communications, labor markets, and international organizations—can inform public policy, address concerns about fairness and trust, and help businesses manage the implications of AI integration. By systematically examining LLM usage across diverse domains, our analysis provides crucial context for policymakers, organizational leaders, and the public to better navigate the benefits, challenges, and societal implications of integrating generative AI into everyday communication.

## Teaser

This study explores AI-generated writing adoption across consumer complaints, corporate press releases, job postings, and international organization communications, revealing widespread use following ChatGPT’s release. Findings highlight significant generative AI integration by small firms, corporations, and global institutions, emphasizing its transformative impact on communication practices, business strategies, and public policy.

## Introduction

The emergence of large language models (LLMs) marked a significant moment in artificial intelligence (AI), offering unprecedented capabilities in natural language processing and generation. This rapid proliferation of LLMs generated both excitement and concern. On the one hand, LLMs have the potential to greatly enhance productivity; in the writing space specifically, they can democratize content creation (especially for non-native speakers). On the other hand, policymakers fear an erosion of trust, risks of biases and discrimination, and job displacement^1^^,^^2^^,^^3^; businesses worry about reliability and data privacy; academics debate the implications for research integrity and teaching^4^^,^^5^; and the public is concerned about misinformation, deepfakes, and authenticity.^6^^,^^7^ Further complicating the discourse is the question of how LLMs may widen or potentially bridge socioeconomic gaps, given differential access to these advanced technologies.

Although some early adoption stories or isolated examples have drawn significant media attention and survey studies have explored LLM adoption from an individual user perspective,^8^^,^^9^ there remains a lack of systematic evidence about the patterns and extent of LLM adoption across various diverse writing domains. While some previous work used commercial software to detect such patterns,^10^^,^^11^ these studies have often been constrained to single domains, relied on black-box commercial AI detectors, or analyzed relatively small datasets. To address this gap, we conduct a large-scale, systematic analysis of LLM adoption patterns across consumer, firm, and institution communications. Our analysis leverages a statistical framework validated in our previous work^12^ to quantify the prevalence of LLM-assisted writing. For the purposes of this study, “LLM-assisted” refers to text that has been entirely generated or substantially modified by LLMs, beyond simple spelling or grammar fixes. Such assistance may include summarizing existing content or generating prose from structural outlines.

We focus on four domains where LLMs are likely to influence communication and decision-making: consumer complaints, corporate press releases, job postings, and United Nations (UN) press releases. Consumer complaints offer insight into user-business interactions and show how these technologies may extend beyond AI-powered customer service.^13^ Corporate press releases reflect strategic organizational usage as firms incorporate LLMs into their investor relations, public relations (PR), and broader business communications. Job postings reveal how recruiters and human resource departments harness LLMs, shedding light on broader labor market trends. Finally, UN press releases showcase the growing institutional adoption of AI for regulatory, policy, and public outreach efforts. We also conducted a similar analysis of patent applications. However, due to the standard 18-month embargo between application and publication, our study period did not yield sufficient data to draw robust conclusions. Still, in the limited sample of late-2024 published patents, we observed a (very) moderate uptick in LLM-assisted text. The job postings and consumer complaints originate from US sources, while the corporate and UN press releases have international coverage but are restricted to English-language content.

This comprehensive approach reveals several patterns. First, we observe a consistent trajectory across all the analyzed domains: rapid initial adoption following ChatGPT’s release, followed by a distinctive stabilizing trend highlighting widespread adoption. We observe that adoption patterns are similar across these diverse domains. By the end of the period we analyzed, in the financial dataset, we estimate that about 18% of the data were LLM assisted, around 24% in company press releases, up to 15% for young and small company job postings, and 14% for international organizations. Second, we uncover some heterogeneity in adoption rates across geographic regions, demographic groups, and organizational characteristics. Third, we find that organizational age and size emerge as the most important predictors of differential adoption, with smaller and younger firms showing markedly higher utilization rates.

Our findings provide insights into the early evidence of LLM integration across society, revealing how various socioeconomic and organizational factors shape technology adoption patterns. This understanding is essential for policymakers, business leaders, and researchers as they navigate the implications of AI integration across different sectors of society and work to ensure equitable access to and responsible deployment of these powerful new tools in the future.

## Results

### Widespread adoption of LLMs in writing assistance across domains

**Figure 1:**
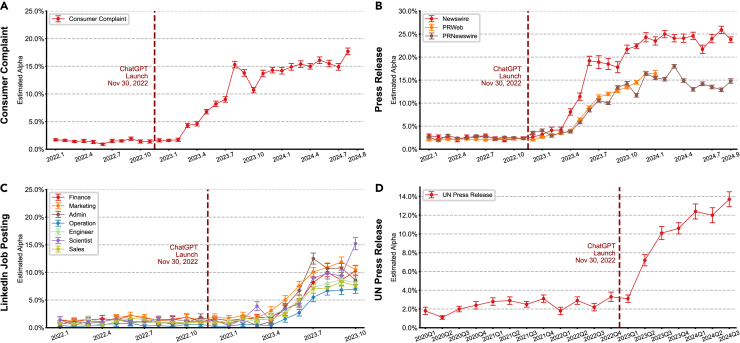
Temporal dynamics of large language model adoption across diverse writing domains Analysis of large language model (LLM)-assisted content across four domains. (A) Consumer complaints filed with the Consumer Financial Protection Bureau showed algorithm false positive rate of 1.5% pre-ChatGPT release (November 2022), followed by genuine LLM adoption rising to 15.3% by August 2023, before plateauing at 17.7% through August 2024. (B) Corporate press releases demonstrated consistent adoption patterns across platforms: Newswire platform showed rapid uptake reaching 24.3% by December 2023 and stabilizing at 23.8% through September 2024; PRNewswire demonstrated similar trends with peak adoption at 16.4% (December 2023) and maintaining at 16.5% through September 2024; and PRWeb showed comparable patterns (data available through January 2024). See also Figure S1. (C) LinkedIn job postings from small organizations (below median job postings) displayed consistent trends across professional categories, with adoption increasing post-ChatGPT release (5-month lag) and peaking in July 2023 before plateauing or slightly declining through October 2023. See also Figures S2 and S3. (D) United Nations government press releases showed two phases: rapid initial adoption (Q1 2023: 3.1% to Q3 2023: 10.1%), followed by a more gradual increase to 13.7% by Q3 2024. See also Figure S4. This figure displays the fraction (*α*) of sentences estimated to be LLM assisted using our previous method.^12^ Error bars indicate 95% confidence intervals by bootstrap. Data are presented as mean ± 95% confidence interval (CI) based on 1,000 bootstrap iterations.

We systematically analyzed LLM adoption patterns across four distinct domains: consumer complaints, corporate PR communications, job postings, and governmental press releases (see methods for data collection and preprocessing). Our analysis reveals a consistent pattern of initial rapid adoption following ChatGPT’s release, followed by a notable stabilization period that emerged between mid- to late 2023 across all domains (Figure 1). While the patterns across all time series show a slower adoption through 2024, these could be (at least partly) the product of more sophistication when adopting AI tools or the developments in LLMs making writing more indistinguishable from human writing.

In the consumer complaint domain (Figure 1A), initial LLM adoption surged about 3–4 months after the release of ChatGPT in November 2022. The proportion of content flagged as LLM assisted rose sharply from a baseline algorithm false positive rate of 1.5% to a rate of 15.3% by August 2023. This rapid growth plateaued, with only a modest increase to 17.7% observed through August 2024.

Corporate press releases demonstrated similar adoption trends across platforms (Figure 1B), once again about 3–4 months post-ChatGPT release. Newswire saw rapid growth, peaking at 24.3% in December 2023 and stabilizing at 23.8% through September 2024. PRNewswire followed closely, reaching 16.4% in December 2023 and maintaining this level through September 2024. PRWeb exhibited comparable dynamics, with data available through January 2024.

**Figure 2:**
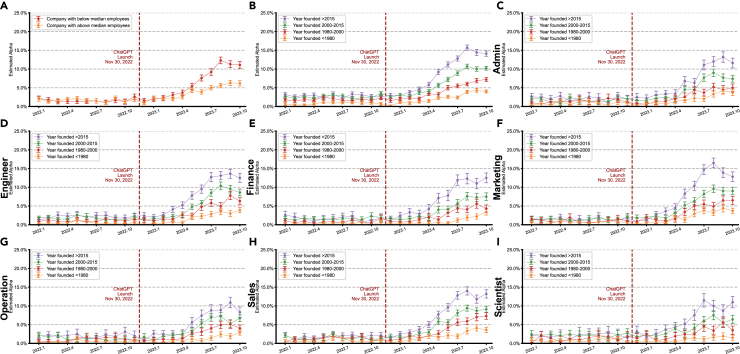
Organization age and LLM adoption patterns in LinkedIn job postings from small organizations across professional categories (A) Among small organizations (less than median job vacancies), analysis stratified by number of employees revealed higher LLM adoption rates in firms with below median employees (11.1% versus 6.2% by October 2023). (B) Among small organizations (less than median job vacancies), analysis stratified by founding year revealed higher LLM adoption rates in more recently established firms (founded after 2015: 14.1%; 2010–2015: 10.2%; 1980–2000: 7.2%; and pre-1980: 4.0%). (C–I) This age-dependent pattern persisted across professional categories: admin (C), engineering (D), finance (E), marketing (F), operations (G), sales (H), and scientist (I), with newer organizations consistently showing higher adoption rates. We defined small organizations based on having 2 or fewer job vacancy postings in a year (median is 3). Data are presented as mean ± 95% CI based on 1,000 bootstrap iterations.

LinkedIn job postings from small organizations showed profession-specific adoption trends but similarly reflected a slowing trajectory (Figure 1C). Following a 5-month lag post-ChatGPT release, adoption increased steadily across professional categories, peaking in July 2023 between 6% and 10%. These figures are higher in the sample of small and young firms, where they reach more than 10% and up to 15% (Figure 2). Adoption rates either plateaued or showed signs of slight declines through October 2023, when the latest data were available.

International organization communication, here measured by UN press releases by country teams, followed a similar adoption pattern with initial rapid growth that later plateaued (Figure 1D). The initial phase was marked by a rapid increase from 3.1% in Q1 2023 to 10.1% in Q3 2023. This was followed by a slower, incremental rise, reaching 13.7% by Q3 2024.

### Geographic and demographic disparities in consumer complaint LLM adoption

**Figure 3:**
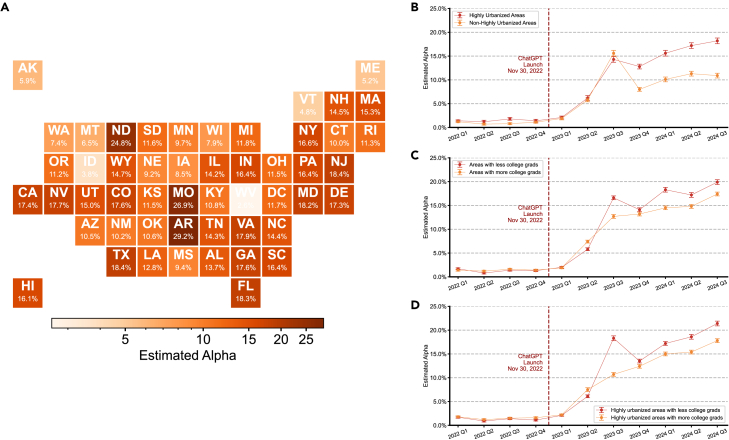
Geographic and demographic patterns of LLM adoption in Consumer Financial Protection Bureau complaints (A) State-level analysis (January–August 2024) revealed substantial geographic variation, with highest adoption in Arkansas (29.2%), Missouri (26.9%), and North Dakota (24.8%), contrasting with lowest rates in West Virginia (2.6%), Idaho (3.8%), and Vermont (4.8%). Notable population centers showed moderate adoption (California: 17.4%, New York: 16.6%). (B) Analysis by rural urban commuting area (RUCA) codes showed similar adoption trajectories between highly urbanized and non-highly urbanized areas during initial uptake (Q1–Q3 2023) before diverging to equilibrium levels of 18.2% and 10.9%, respectively. (C) Comparison of areas above and below state median levels of bachelor’s degree attainment (population aged 25+) revealed comparable initial adoption patterns (Q1–Q2 2023), followed by higher stabilized rates in areas with lower educational attainment (19.9% versus 17.4% by Q3 2024). (D) Within highly urbanized areas, this educational attainment pattern persisted, with lower-education areas showing higher adoption rates (21.4% versus 17.8% by Q3 2024). Data are presented as mean ± 95% CI based on 1,000 bootstrap iterations.

Our analysis of Consumer Financial Protection Bureau (CFPB) complaints revealed some geographic and demographic heterogeneity in LLM adoption patterns (Figure 3). At the state level, we observed variation in adoption rates during the January–August 2024 period, with the highest adoption in Arkansas (29.2%, 7,376 complaints), Missouri (26.9%, 16,807 complaints), and North Dakota (24.8%, 1,025 complaints). This contrasted sharply with minimal adoption in West Virginia (2.6%, 2,010 complaints), Idaho (3.8%, 1,651 complaints), and Vermont (4.8%, 361 complaints). Notably, major population centers demonstrated much less variation in adoption levels, with California (157,056 complaints) and New York (104,862 complaints) showing rates of 17.4% and 16.6%, respectively (Figure 3A). However, this could be interpreted either as a genuine differential compared to the smaller states in the left and right tails or as the product of lower sample noise (due to higher numbers of observations).

The adoption of LLMs varied over time between more and less urbanized areas. Analysis using rural-urban commuting area (RUCA) codes showed that highly urbanized and non-highly urbanized areas initially displayed similar adoption trajectories during the early phase (Q1–Q3 2023). However, these trajectories subsequently diverged, reaching equilibrium levels of 18.2% in highly urbanized areas compared to 10.9% in non-highly urbanized areas (Figure 3B). These differences were highly statistically significant at all conventional levels.

Areas with lower educational attainment showed somewhat higher LLM adoption rates in consumer complaints. Comparing areas above and below the state median levels of bachelor’s degree attainment, areas with lower educational attainment ultimately stabilized at rates of around 19.9% in Q3 2024 (compared with 17.4%) (Figure 3C). This pattern persisted even within highly urbanized areas, where lower-education regions demonstrated higher adoption rates (21.4% versus 17.8% by Q3 2024) (Figure 3D). In both comparisons, the *p* values were less than 0.001, indicating statistically significant differences, despite qualitatively similar trends.

### LLM adoption in corporate press releases

**Figure 4:**
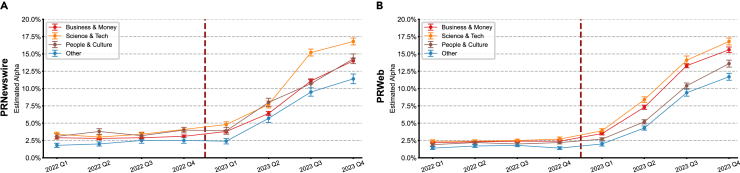
Sectoral patterns of LLM adoption in corporate press releases across major distribution platforms Analysis of press releases by sector revealed consistent patterns across platforms, with science and technology showing marginally higher adoption rates. (A) PRNewswire demonstrated similar sectoral patterns by Q4 2023: science and technology (16.8%), people and culture (14.3%), business and money (14.0%), and other sectors (11.4%). (B) PRWeb exhibited comparable sectoral distribution: science and technology (16.8%), business and money (15.6%), people and culture (13.6%), and other sectors (11.7%). All sectors showed similar temporal adoption patterns following ChatGPT’s release, with an initial lag followed by sustained growth through 2023. Data are presented as mean ± 95% CI based on 1,000 bootstrap iterations.

After characterizing consumer-side adoption patterns, we next examined corporate LLM usage across major corporate press release platforms—Newswire, PRWeb, and PRNewswire, each of which caters to different audiences and industries (Figures 1B, 4A, 4B, and S1). A vast oversimplification based on available data would be that PRNewswire generally targets larger corporations with extensive reach to major news outlets and traditional media. PRWeb offers a more affordable, online-focused option with an emphasis on SEO, catering to smaller businesses. Newswire reaches both traditional and online platforms. All three offer some editorial services but focus primarily on the distribution of the content produced by the businesses.

Before the launch of ChatGPT, the fraction of LLM-assisted content remained consistently low across all these sources, fluctuating around the 2%–3% mark (i.e., false positives). However, following the launch, a significant increase in LLM-assisted content was observed across all domains, about two quarters post-rollout. Newswire, in particular, experienced the most dramatic rise, with the estimated fraction peaking at over 25% by late 2023. PRWeb and PRNewswire also saw notable growth, though to a lesser degree, plateauing around 15%. This suggests a widespread uptake of LLM technology in content creation across different types of press releases starting in early 2023.

In Figures 4A and 4B, we show the quarterly growth of LLM usage in press releases across different categories for PRNewswire (Figure 4A) and PRWeb (Figure 4B). Both charts show a sharp rise in LLM-assisted content starting in early 2023, with some differential patterns emerging by topic. In both platforms, the categories “business and money” and “science and technology (tech)” exhibit the most pronounced increase, with science and tech reaching just below 17% by Q4 2023. “People and culture” and “other” categories also demonstrate growth but at a somewhat slower pace, which may be indicative that LLM adoption has been particularly strong in more technical and business-focused content.

Overall, we show a significant uptick in LLM-assisted writing across various press release categories. On the one hand, the sharp increase in LLM-assisted content in press releases suggests that businesses are leveraging LLMs to improve efficiency in content creation. By utilizing AI, companies can potentially produce communications more quickly and cost effectively, especially in areas requiring frequent updates and complex information dissemination. This may also be advantageous if companies are trying to withhold more sensitive information from the public and use more generic language. On the other hand, the growing reliance on LLM-assisted content may introduce challenges in communication. In sensitive categories, over-reliance on AI could result in messages that fail to address concerns or overall release less credible information externally. Over-reliance on AI could also introduce public mistrust in the authenticity of messages sent by firms.

### LLM adoption in LinkedIn job postings

We next examined another dimension of corporate LLM adoption through analysis of LinkedIn job postings. We first took the whole sample of LinkedIn job postings and analyzed the effects (Figure S2). In this full sample, we see that about 3%–4% of all vacancy postings have LLM-assisted content. Although this is a small increase, it is generally statistically different from pre-ChatGPT introduction (i.e., false positive) levels (with *p* values less than 0.001 across categories). However, this broader sample heavily features larger firms that post more vacancies and have greater financial and human resources to customize those postings. Such firms may also advertise the same position multiple times throughout the year and rely on their established reputation, reducing the need to update job postings frequently. Consequently, for the remainder of this analysis, we focused on small companies, defined either as firms that post fewer than the median number of vacancies (2 or fewer each year) or as businesses with 10 or fewer registered employees in 2021 or those posting two or fewer positions per year on LinkedIn.

Using the sample of small companies based on the number of vacancies posted, our findings reveal a gradual but notable increase in the estimated fraction of LLM-assisted content for several job categories (Figures 1D and 2). Prior to the launch of ChatGPT, the fraction of LLM-assisted text hovered between 0% and 2% across all categories, reflecting the range of false positives. After ChatGPT became available, a discernible uptick began around early to mid-2023, leveling off by October 2023 at roughly 5%–10% for all categories. The increase is most pronounced in engineering and sales postings, which each approach 10% LLM-assisted content. Finance, administration (admin), scientist, and operations categories show a somewhat slower growth, albeit the differences between them are small. If instead we define small companies by the number of employees (Figure S3), the scientist category ranks first. This may be some evidence that firms requiring more advanced scientific, financial, or marketing expertise might be more inclined to adopt AI technologies, although the differences are modest.

We further stratified these small firms by founding year—grouping them into post-2015, 2000–2015, 1980–2000, and pre-1980 cohorts (Figure 2), based on the rough quartiles in the data. Across every job category, more recently founded companies consistently exhibit both the highest levels and the fastest uptake of LLM-related text generation, especially following ChatGPT’s launch. Firms founded after 2015 reached 10%–15% LLM-assisted text in certain roles, whereas those founded between 2000 and 2015 showed moderate growth of 5%–10%. By comparison, firms founded before 1980 typically remained below 5%. These results underscore how younger firms—possibly with younger workforces—more readily integrate new AI technologies into their hiring and onboarding processes, whereas older organizations may adopt such tools more conservatively. Overall, firm age and size emerge as (perhaps the most) significant correlates of the heterogeneity observed in LLM uptake throughout our analyses.

This trend highlights a potential shift in recruitment practices among small firms, showcasing a growing reliance on AI writing tools. On the one hand, this can decrease company hiring costs, with smaller and younger enterprises being more likely to leverage advanced tools to remain competitive despite perhaps being more liquidity constrained. On the other hand, the adoption of LLM writing in job postings could either enhance or decrease the efficiency and effectiveness in attracting qualified candidates. For job seekers, one possible negative effect is having a harder time differentiating between the posting firm’s quality and position requirements.

The leveling off or even slight decrease in LLM-assisted content by October 2023 might indicate that the adoption rate has stabilized, potentially reaching a saturation point where firms that are comfortable with AI have already adopted it. Alternatively, this can be explained by the increased sophistication and subtlety of these methods. Overall, the increased integration of AI in job postings suggests a transformative period in hiring, with AI playing an important role in how small firms communicate job opportunities. This could have implications for job seekers as well, who may encounter more uniformly crafted postings and might need to adapt their application strategies accordingly.

### LLM adoption in UN press releases

UN press releases exhibited a similar two-phase adoption pattern, with an initial surge from 3.1% to 10.1% in Q1–Q3 2023, followed by a more gradual increase to 13.7% by Q3 2024 (Figure 1D). Across UN member states’ country teams, we observed consistent adoption patterns across regions, with adoption rates reaching 11%–14% by 2024, with the exception of the UN teams in Latin American and Caribbean countries that had slightly higher adoption rates at about 20% (Figure S4). The steady growth across regions reflects how LLMs are being integrated globally, even in contexts of sensitive, high-stakes communication.

This rapid uptake suggests that country teams have found LLMs valuable for producing timely updates, which can be especially useful during pressing crises. On the other hand, this trend raises questions about how LLMs might affect the authenticity of vital international communication. As the UN continues to refine its stance on AI, this highlights a broader trend: even the world’s most prominent international bodies are using LLMs in their communications—underscoring both the perhaps inevitability of AI-driven writing and the questions it raises about authenticity and accountability.

## Discussion

In this section, we relate three key empirical findings to existing literature, discuss methodological limitations, propose directions for future research, and conclude.

### Cross-sector adoption of LLM-assisted writing

First, our analysis reveals widespread adoption of LLMs across diverse writing domains, including consumers, corporations, and international organizations. By late 2024, roughly 18% of financial consumer complaint texts appear to be LLM assisted, up to 24% of corporate press releases, and just below 10% in small firm job postings. Even UN press releases reflect this trend, with nearly 14% of the text estimated to be LLM assisted. The overall trend and presence of LLM-assisted content suggest that AI-driven writing tools have a broad and expanding reach. This finding complements and extends our previous research that found widespread adoption across academic researchers.^12^

This pattern reflects a broader transformation in writing and content production. Media and communication scholars have long studied how technologies like printing, word processors, and digital platforms, such as social media, reshape writing and content creation practices. These changes have often prompted debate over whether expanding access to technologies enables broader participation and creativity or undermines the role of expertise, authorship, and quality control.^14^^,^^15^^,^^16^ Our findings suggest that LLMs continue this trajectory—making writing and content creation more accessible while raising concerns about uniformity, authorship, and editorial accountability.

This cross-sector adoption of LLM-assisted writing also relates to ongoing debates in the economics literature about AI’s broader impact on productivity and growth. Estimates of AI’s contribution to gross domestic product (GDP) vary widely, from under 1% to over 7% over the next decade,^17^^,^^18^ depending in part on which tasks are automatable, the pace of adoption, and realized productivity gains. Writing is often cited as an early domain of impact, given its ubiquity and cost.^19^^,^^20^ Our findings suggest that consumers, firms, and institutions are already incorporating LLMs into written communication at scale. Future research should assess whether this trend translates into meaningful productivity improvements, including whether time saved through LLM use^21^ enables reallocation of effort toward higher-value writing tasks or other forms of knowledge work.

### Temporal diffusion patterns in LLM-assisted writing

Second, our data reveal a distinct temporal pattern in LLM-assisted writing: following an initial lag of 3–4 months after the launch of ChatGPT, usage increased sharply, then stabilized by late 2023, and remained mostly steady through 2024. While this trajectory is broadly consistent with the classical “S curve” of technology adoption—where uptake begins slowly, accelerates with early majority adoption, and eventually levels off^22^^,^^23^^,^^24^—the early plateau we observe raises questions about the pace and scope of broader diffusion.

Several factors may help explain this pattern. One possibility is that adoption has reached saturation among early adopters but has yet to expand into wider segments of the population. Alternatively, the plateau may reflect structural frictions that inhibit further uptake. We identify three categories of potential barriers: institutional, technological, and perceptual. Institutional frictions may stem from entrenched business practices or organizational inertia, particularly in older or more hierarchical institutions.^25^ This could help account for the relatively lower adoption observed among larger firms. Regulatory constraints may also play a role, as seen in sectors like healthcare, where AI integration faces compliance hurdles.^26^ Technological limitations could persist as well. Despite ongoing improvements, LLMs continue to produce hallucinations and factual errors,^27^ limiting their applicability in high-stakes environments. Finally, perceptual frictions may emerge when audiences detect and react negatively to AI-generated content. Prior research suggests that such writing is often perceived as less trustworthy or authentic,^28^^,^^29^^,^^30^ which may reduce its acceptance in public-facing communication.

### Socio-demographic patterns in LLM-assisted writing

Third, the geographic and demographic patterns in LLM adoption also present an intriguing departure from historical technology diffusion trends^24^^,^^31^ and technology acceptance models,^32^^,^^33^ where technology adoption has generally been concentrated in urban areas, among higher-income groups, and in populations with higher levels of educational attainment.^34^^,^^35^ While the urban-rural digital divide seems to mostly persist, we found that areas with lower educational attainment showed modestly higher LLM adoption rates in consumer complaints. This suggests that AI writing tools may serve as equalizing tools in consumer advocacy. This finding aligns with survey evidence indicating that younger, less experienced workers may be more likely to use ChatGPT.^8^ This democratization of access underscores the potentially transformative role LLMs could play in amplifying underserved voices. However, further study is needed to assess whether this increased adoption translates into more effective consumer outcomes.

Beyond these three main findings, our results also reveal additional noteworthy patterns. In the recruitment process, we saw many companies, especially small and younger firms, producing LLM-assisted job postings. This may superficially seem in contrast with data points showing that larger and more liquid firms increase their AI investments more.^36^ We think it is possible that younger, smaller firms may use more cost-effective AI products, such as ChatGPT, that are easier to capture in our data and may also have a shorter time from usage to output. While our study did not directly measure the homogenization of posting between firms, prior research on the homogenization of LLM-assisted content in academia^12^^,^^37^ suggests that similar effects could occur in recruitment. Homogenization may inadvertently obscure critical distinctions between roles and organizations, potentially complicating job seekers’ decision-making. In fact, recent evidence has shown that while employers who leverage LLM to generate the first draft of a job post may receive more applications, they are less likely to make a hire.^38^ Further investigating how these postings influence applicant perceptions and hiring could provide valuable insights into the long-term implications of this shift.

### Limitations and future work

Our study has several limitations. While we focused on widely used LLMs like ChatGPT, which account for a significant portion of global usage,^39^ we acknowledge that other models also contribute to content generation across domains. Additionally, although prior research has shown that GPT-detection methods can sometimes misclassify non-native writing as AI generated,^40^ our findings consistently indicated low false positive rates during earlier periods. However, shifts in user demographics or language usage could still influence detection accuracy.^41^

Perhaps the biggest limitation in our study is that we cannot reliably detect language that was generated by LLMs but was either heavily edited by humans or generated by models that imitate human writing very well. Therefore, one way to interpret our study is as a lower bound of adoption patterns. Finally, our analysis primarily focuses on English-language content, potentially overlooking adoption trends in non-English-speaking regions. Future research could expand on these findings by incorporating multilingual data and refining detection methodologies.

In conclusion, our findings suggest that LLM writing has become increasingly common across consumer, corporate, recruitment, and even governmental communications. As these technologies continue to mature, understanding their effects on content quality, creativity, and information credibility will be critical. Addressing the regulatory and ethical challenges associated with LLM-assisted content will also be essential for ensuring that the benefits of LLMs are realized while maintaining transparency, diversity, and public trust in communication.

## Methods

### The distributional LLM quantification framework

We adapt the distributional LLM quantification framework from Liang et al.^12^ to quantify the use of LLM-assisted writing. A key feature of the framework is its population-level perspective, which enables corpus-wide quantification without relying on the identification of individual instances. This framework models the target corpus as a mixture of human-written and LLM-assisted texts. It estimates the share of LLM-assisted content by fitting the empirical frequency of every token (i.e., how often each word appears in the corpus) to reference distributions from human-written and LLM-assisted texts via maximum-likelihood estimation. Put simply, we use lexical differences between LLM texts (see Figures S5 and S6 for details) and known human-written texts (before ChatGPT release) in a domain at different points in time. As validated in a prior paper, the framework has demonstrated robustness, transparency, and low cost,^12^^,^^37^^,^^40^ allowing us to track adoption trajectories and key demographic and organizational factors driving LLM integration.

### Overview of the consumer complaint data

The Consumer Complaint Database, maintained by the CFPB, is a publicly accessible resource that collects complaints about consumer financial products and services. These complaints are forwarded to companies for their response, while the CFPB—a US government agency—is dedicated to ensuring that banks, lenders, and other financial institutions treat consumers fairly. We focus on 687,241 consumer complaint narratives, starting from January 2022 and ending in August 2024. The dataset offers the mailing ZIP code provided by the consumer, which allows us to check heterogeneity via the educational level and the degree of urbanization by region. Specifically, we employ RUCA codes to assess urbanization levels and measure the educational level by the percentage of individuals aged 25 and older who have earned a bachelor’s degree. Corresponding data are available at https://www.ers.usda.gov/data-products/rural-urban-commuting-area-codes and https://data.census.gov/table/ACSST1Y2023.S1501, respectively.

### Overview of the LinkedIn job posting data

We use data from the Revelio Labs universe, which collects, cleans, and aggregates individual-level job postings sourced from publicly available online sources, such as LinkedIn. The raw dataset includes all LinkedIn postings (active, inactive, and removed), the company identifier, the company founding year, the full text of the job listings, and associated information (title, salary, etc.). The raw data are broken out by Revelio Labs into eight job categories: admin, engineering, finance, marketing, operations, sales, scientist, and unclassified. We focus on 304,270,122 job postings, starting from January 2021 and ending in October 2023. We focus on the full text of the job postings. To analyze the heterogeneity of LLM usage by company characteristics, we combine the job listings information with the Revelio Labs-associated LinkedIn employee data. Similarly to the job postings data, the baseline workforce data were scraped, cleaned, and aggregated at the firm level. The workforce data are available going back to 2008. We define firm characteristics based on pre-ChatGPT introduction characteristics. We define two different definitions for small firms: in our sample, small firms are companies with either 10 or fewer registered employees in 2021 or companies posting less than or equal to about 2 postings per year. We also check heterogeneity via founding year, splitting in terms of years 2015 onwards, 2000–2015, 1980–2000, and before 1980. These time periods are determined based on quantiles of the founding year distribution. Note that although the median number of postings per company per year is 3, the total number of postings drops from 304,270,122 to 1,440,912 when we focus on small companies. This indicates that small companies contribute a relatively minor share to the total posting volume compared to larger companies.

### Overview of the corporate press release data

We collect corporate press release data using the NewsAPI service, which aggregates online news content from various sources. We collected data from PRNewswire, PRWeb, and Newswire, three of the main companies distributing corporate press releases online. These were chosen due to data availability and cost. PRNewswire, founded in 1954, is one of the oldest and most widely recognized press release distribution services, offering an extensive network that reaches major news outlets, journalists, and online platforms worldwide. It serves a broad range of clients, from large corporations to small businesses. PRWeb, launched in 1997, focuses primarily on online distribution and SEO optimization, making it a more budget-friendly option for businesses looking to enhance their digital presence. Newswire distributes press releases to both traditional media and online platforms, catering to businesses of various sizes. While all three services offer some level of editorial support, their primary business focus remains distribution.

With a focus on English-language text, we gathered up to 537,413 press releases from January 2022 to September 2024. Our analysis primarily focused on the full body text. Due to the limited number of articles available from Newswire post-ChatGPT introduction, we conducted detailed robustness checks only on PRNewswire and PRWeb data, which provided sufficient volume for heterogeneity analysis. We classified the press releases by four overarching categories: business and money, science and tech, people and culture, and other.

### Overview of the UN press release data

We collect UN release data using customized scripts. The UN, founded in 1945, is an international organization dedicated to fostering global peace, security, and cooperation among its member states.^11^ Country teams of the UN regularly update on the latest developments in that country. To ensure consistency and maintain a focus on English-language content, articles were selected from the English-language websites of 97 country teams. From January 2019 to September 2024, up to 15,919 press releases were collected, with the analysis primarily concentrating on the full body text. Our investigation revealed that among the remaining 96 country teams, 57 do not have their own websites, 33 lack English-language websites, and 6 do not operate press release websites.

### Data split, model fitting, and evaluation

For model fitting, we count word frequencies for the corpora written before the release of ChatGPT and the LLM-assisted corpora. We fit the model with data from 2021 (2019 for UN press releases) and use data from January 2022 onwards for validation and inference. We developed individual models for each major category in LinkedIn job postings and for each distribution platform in corporate press releases. For UN press releases and consumer complaints, we fit one model for each domain. During inference, we randomly sample up to 2,000 records per month (per quarter for UN press release) to analyze the increasing temporal trends of LLM usage across various writing domains.

To evaluate model accuracy and calibration under temporal distribution shift, we collected a sample of 2,000 records from January 1, 2022, to November 29, 2022, a time period prior to the release of ChatGPT, as the validation data. We construct validation sets with LLM-assisted content proportions (*α*) ranging from 0% to 25%, in 2.5% increments, and compared the model’s estimated *α* with the ground-truth *α* (Tables S1–S5). Our models all performed well in our application, with a prediction error consistently less than 3.3% at the population level across various ground-truth *α* values.

## Resource availability

### Lead contact

Requests for further information and resources should be directed to and will be fulfilled by the lead contact, James Zou (jamesz@stanford.edu).

### Materials availability

This study did not generate new unique reagents.

### Data and code availability

• The datasets analyzed in this study are publicly or privately accessible through the following sources: the consumer complaint data are available at the CFPB’s website (https://www.consumerfinance.gov/data-research/consumer-complaints/),^42^ the corporate press release data can be accessed via NewsAPI (https://www.newsapi.ai/),^43^ and LinkedIn job posting data were obtained from the Revelio Labs workforce data, licensed through the Stanford GSB Library (https://www.data-dictionary.reveliolabs.com/).^44^ Data for UN press releases were directly scraped from official UN websites (e.g., https://china.un.org/en/press-centre/press-releases).^45^
• Our source code is available at GitHub (https://github.com/Weixin-Liang/LLM-widespread-adoption-impact) and has been archived at Zenodo.^46^

## Acknowledgments

We thank Daniel McFarland, Dan Jurafsky, Yian Yin, Mirac Suzgun, Zachary Izzo, and Diane Coyle for their helpful comments and discussions. We thank Todd Hines and the Stanford GSB Library for help with obtaining data. J.Z. is supported by the 10.13039/100000001National Science Foundation (CCF 1763191 and CAREER 1942926), the US National Institutes of Health (P30AG059307 and U01MH098953), and grants from the Silicon Valley Foundation and the 10.13039/100014989Chan Zuckerberg Initiative.

## Author contributions

W.L. and Y.Z. designed the study and oversaw the quantification analysis. W.L., Y.Z., and M.C. provided the code for data analysis and conducted the analysis. W.L., Y.Z., M.C., and H.C. wrote the paper, with substantial input from all authors. All authors contributed to the review and editing of the paper. J.Z. provided the overall direction and planning of the project.

## Declaration of interests

The authors declare no competing interests.

## Supplemental information

Document S1. Figures S1–S6 and Tables S1–S5

Document S2. Article plus supplemental information
