# Supplementary material for: The widespread adoption of large language model-assisted writing across society
Source: Patterns (N Y). 2025 Oct 2;6(12):101366. doi: 10.1016/j.patter.2025.101366 (PMC12745980; doi:10.1016/j.patter.2025.101366)
Supplement: Document S1. Figures S1–S6 and Tables S1–S5 [file mmc1.pdf]

Patterns, Volume *6*

Supplemental information

The widespread adoption of large language model-assisted writing across society

Weixin Liang, Yaohui Zhang, Mihai Codreanu, Jiayu Wang, Hancheng Cao, and James Zou

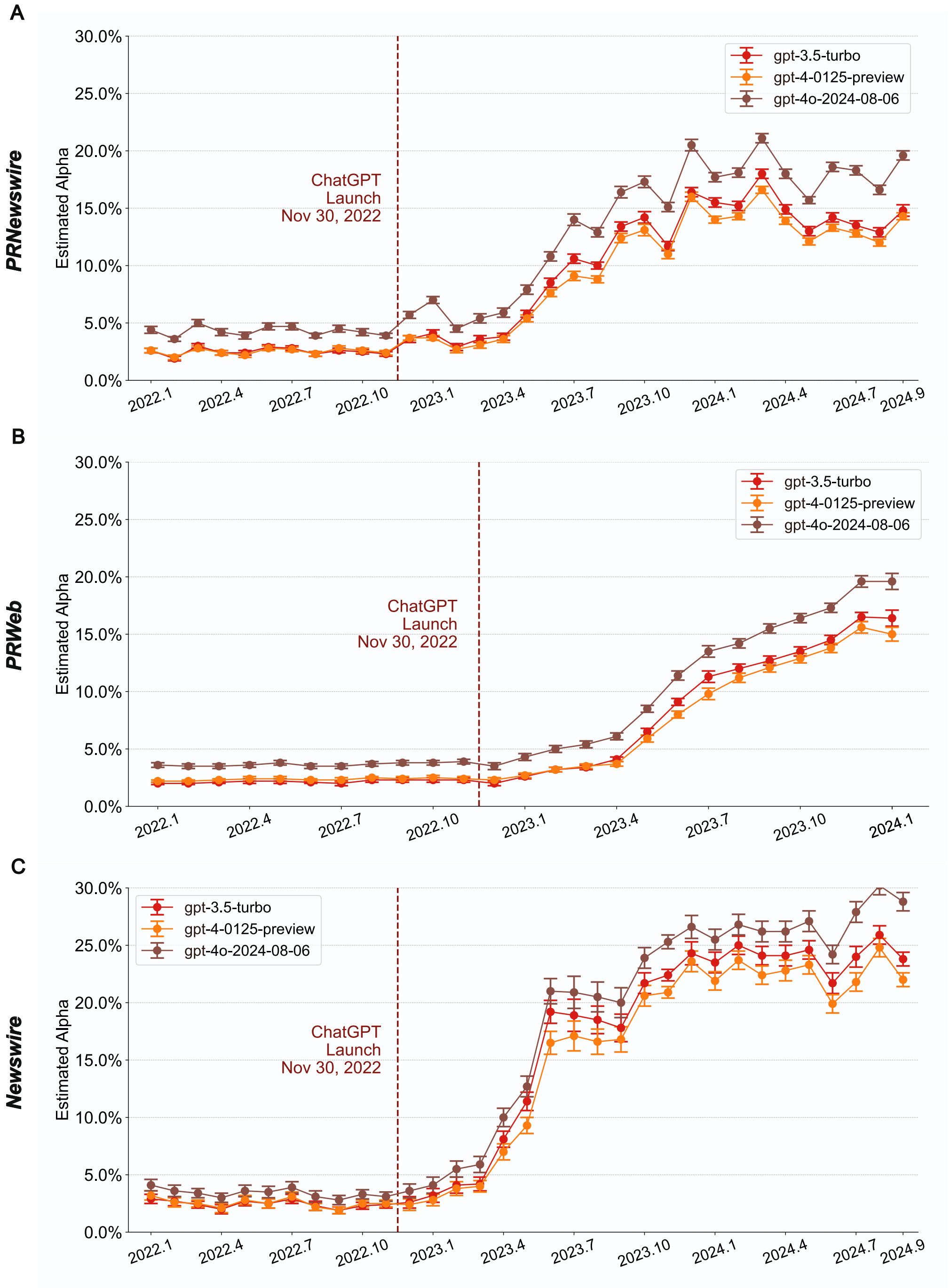

Figure S1. **Robustness analysis of LLM adoption estimates across different press release platforms using multiple GPT models for training data generation**, related to Figure 1. (A) PRNewswire, (B) PRWeb, and (C) Newswire press releases show consistent temporal patterns regardless of the GPT model used for training data generation. Estimated fraction (α) of LLM-assisted content was calculated using three different models: GPT-3.5-turbo (used in main analysis, released January 25, 2024), GPT-4-0125-preview (released January 25, 2024), and GPT-4-2024-08-06 (released August 6, 2024). While all models reveal similar adoption trajectories following ChatGPT's launch (November 30, 2022), the most recent model GPT-4-2024-08-06 generates marginally higher estimates across platforms, suggesting our main results may be conservative. Error bars indicate 95% confidence intervals obtained through bootstrap analysis. Data are presented as mean $\pm$ 95% CI based on 1,000 bootstrap iterations.

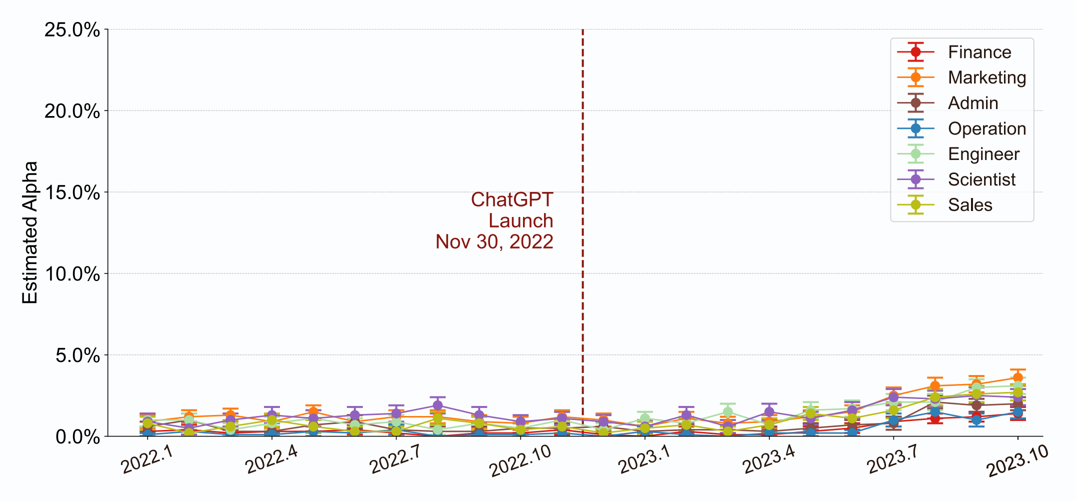

Figure S2. **Analysis of LLM adoption in LinkedIn job postings across the full sample**, related to Figure 1. Temporal analysis of the estimated fraction (α) of LLM-assisted content in job postings across all company sizes shows a modest but statistically significant increase from pre-ChatGPT baseline to approximately 3% adoption following ChatGPT's introduction (November 30, 2022). This aggregate analysis includes all companies regardless of size, with larger firms (who post more frequent vacancies and typically have dedicated HR resources) representing a greater proportion of the sample. Error bars represent 95% confidence intervals obtained through bootstrap analysis. Data are presented as mean $\pm$ 95% CI based on 1,000 bootstrap iterations.

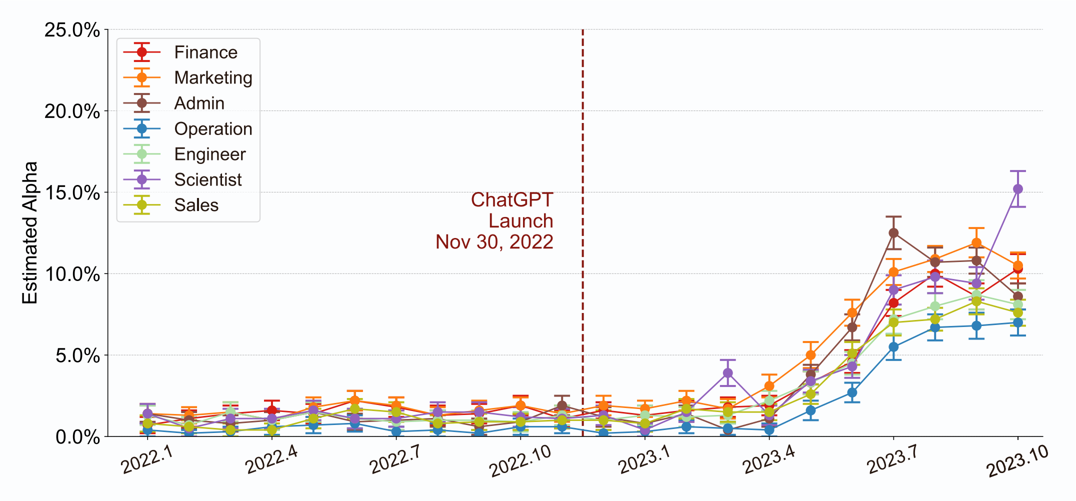

Figure S3. **LLM adoption patterns in LinkedIn job postings from small organizations ($\leq$10 employees)**, related to Figure 1. Temporal analysis of estimated fraction (α) of LLM-assisted content across professional categories (Finance, Marketing, Admin, Operation, Engineer, Scientist, Sales) shows patterns consistent with main findings based on vacancy frequency. Following ChatGPT's launch (November 30, 2022), organizations with $\leq$10 employees demonstrate similar adoption trajectories to those posting $\leq$2 vacancies annually, with estimated α increasing from 0-2% pre-launch to 7-15% by October 2023. Scientist positions show highest adoption ($\approx$15%), followed by Marketing and Finance ($>$10%), while Admin, Engineer, Sales and Operations show more moderate adoption (7-9%). Error bars indicate 95% confidence intervals obtained through bootstrap analysis. This consistency across different definitions of small organizations (by employee count or vacancy frequency) strengthens the robustness of observed adoption patterns. Data are presented as mean $\pm$ 95% CI based on 1,000 bootstrap iterations.

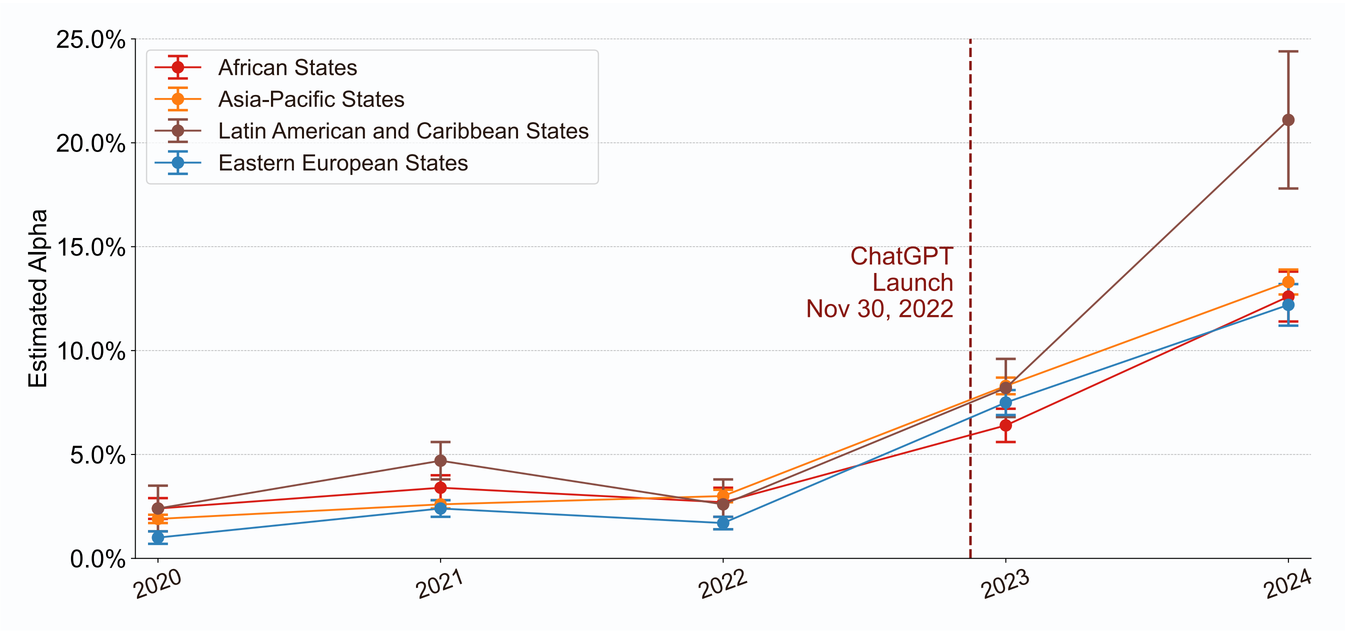

Figure S4. **Regional variation in LLM adoption across United Nations Member States' press releases**, related to Figure 1. Temporal analysis of estimated fraction (α) of LLM-assisted content stratified by regional groups shows differential adoption patterns. After ChatGPT's launch (November 30, 2022), Latin American and Caribbean States demonstrated the highest adoption rate, reaching approximately 21% by 2024, while African States, Asia-Pacific States, and Eastern European States showed more moderate increases to 11-14%. Error bars indicate 95% confidence intervals obtained through bootstrap analysis. Regional variations may reflect differences in technological infrastructure, language diversity, and institutional policies across Member States. Data are presented as mean $\pm$ 95% CI based on 1,000 bootstrap iterations.

```
The aim here is to reverse-engineer the author's writing process by taking a
   piece of text from a consumer complaint and compressing it into a more
   concise form. This process simulates how an author might distill their
   thoughts and key points into a structured, yet not overly condensed form.

Now as a first step, given a complete piece of text from a consumer complaint,
   reverse-engineer it into a list of bullet points.
```

Figure S5. **Example prompt for summarizing a consumer complaint into a skeleton**: This process simulates how an author might first only write the main ideas and core information into a concise outline. The goal is to capture the essence of the complaint in a structured and succinct manner, serving as a foundation for the next prompt.

```
Following the initial step of reverse-engineering the author's writing process
   by compressing a text segment from a consumer complaint, you now enter the
   second phase. Here, your objective is to expand upon the concise version
   previously crafted. This stage simulates how an author elaborates on the
   distilled thoughts and key points, enriching them into a detailed, structured
    narrative.

Given the concise output from the previous step, your task is to develop it into
    a fully fleshed-out text.
```

Figure S6. **Example prompt for expanding the skeleton into a full text**: The aim here is to simulate the process of using the structured outline as a basis to generate comprehensive and coherent text. This step mirrors the way an author might flesh out the outline into detailed paragraphs, effectively transforming the condensed ideas into a fully articulated consumer complaint. The format and depth of the expansion can vary, reflecting the diverse styles and requirements of different consumer complaints.

Table S1. **Performance validation of our model** across Consumer Complaint (all predating ChatGPT's launch), using a blend of official human and LLM-assisted complaints.

No.	Validation Data Source	Ground Truth α	Estimated		Prediction Error
			α	CI $(\pm)$	
(1)	*Consumer Complaint*	0.0%	1.8%	0.2%	1.8%
(2)	*Consumer Complaint*	2.5%	4.6%	0.2%	2.1%
(3)	*Consumer Complaint*	5.0%	7.3%	0.2%	2.3%
(4)	*Consumer Complaint*	7.5%	9.8%	0.2%	2.3%
(5)	*Consumer Complaint*	10.0%	12.2%	0.3%	2.2%
(6)	*Consumer Complaint*	12.5%	14.6%	0.2%	2.1%
(7)	*Consumer Complaint*	15.0%	17.1%	0.3%	2.1%
(8)	*Consumer Complaint*	17.5%	19.4%	0.3%	1.9%
(9)	*Consumer Complaint*	20.0%	21.8%	0.3%	1.8%
(10)	*Consumer Complaint*	22.5%	24.2%	0.3%	1.7%
(11)	*Consumer Complaint*	25.0%	26.5%	0.3%	1.5%

Table S2. **Performance validation of our model** across UN Press Release (all predating ChatGPT's launch), using a blend of official human and LLM-assisted press releases.

No.	Validation Data Source	Ground Truth α	Estimated		Prediction Error
			α	CI $(\pm)$	
(1)	*UN Press Release*	0.0%	2.5%	0.2%	2.5%
(2)	*UN Press Release*	2.5%	5.4%	0.2%	2.9%
(3)	*UN Press Release*	5.0%	8.1%	0.3%	3.1%
(4)	*UN Press Release*	7.5%	10.7%	0.3%	3.2%
(5)	*UN Press Release*	10.0%	13.1%	0.3%	3.1%
(6)	*UN Press Release*	12.5%	15.6%	0.3%	3.1%
(7)	*UN Press Release*	15.0%	18.0%	0.3%	3.0%
(8)	*UN Press Release*	17.5%	20.4%	0.3%	2.9%
(9)	*UN Press Release*	20.0%	22.8%	0.3%	2.8%
(10)	*UN Press Release*	22.5%	25.1%	0.3%	2.6%
(11)	*UN Press Release*	25.0%	27.5%	0.3%	2.5%

Table S3. **Performance validation of our model** across PRNewswire, PRWeb, Newswire (all predating ChatGPT's launch), using a blend of official human and LLM-assisted press releases. Our algorithm demonstrates high accuracy with less than 3.3% prediction error in identifying the proportion of LLM press release within the validation set.

**No.**	**Validation Data Source**	**Ground Truth α**	**Estimated**		**Prediction Error**
			α	CI ($\pm$)	
(1)	*PRNewswire*	0.0%	2.9%	0.3%	2.9%
(2)	*PRNewswire*	2.5%	5.7%	0.3%	3.2%
(3)	*PRNewswire*	5.0%	8.3%	0.3%	3.3%
(4)	*PRNewswire*	7.5%	10.8%	0.3%	3.3%
(5)	*PRNewswire*	10.0%	13.2%	0.3%	3.2%
(6)	*PRNewswire*	12.5%	15.6%	0.3%	3.1%
(7)	*PRNewswire*	15.0%	18.0%	0.3%	3.0%
(8)	*PRNewswire*	17.5%	20.3%	0.3%	2.8%
(9)	*PRNewswire*	20.0%	22.7%	0.3%	2.7%
(10)	*PRNewswire*	22.5%	25.0%	0.3%	2.5%
(11)	*PRNewswire*	25.0%	27.3%	0.3%	2.3%
(12)	*PRWeb*	0.0%	2.1%	0.2%	2.1%
(13)	*PRWeb*	2.5%	5.2%	0.2%	2.7%
(14)	*PRWeb*	5.0%	7.8%	0.2%	2.8%
(15)	*PRWeb*	7.5%	10.4%	0.2%	2.9%
(16)	*PRWeb*	10.0%	12.9%	0.3%	2.9%
(17)	*PRWeb*	12.5%	15.4%	0.3%	2.9%
(18)	*PRWeb*	15.0%	17.8%	0.3%	2.8%
(19)	*PRWeb*	17.5%	20.2%	0.3%	2.7%
(20)	*PRWeb*	20.0%	22.6%	0.3%	2.6%
(21)	*PRWeb*	22.5%	25.0%	0.3%	2.5%
(22)	*PRWeb*	25.0%	27.3%	0.3%	2.3%
(23)	*Newswire*	0.0%	2.3%	0.2%	2.3%
(24)	*Newswire*	2.5%	5.3%	0.2%	2.8%
(25)	*Newswire*	5.0%	7.9%	0.3%	2.9%
(26)	*Newswire*	7.5%	10.5%	0.3%	3.0%
(27)	*Newswire*	10.0%	13.0%	0.3%	3.0%
(28)	*Newswire*	12.5%	15.4%	0.3%	2.9%
(29)	*Newswire*	15.0%	17.9%	0.3%	2.9%
(30)	*Newswire*	17.5%	20.3%	0.3%	2.8%
(31)	*Newswire*	20.0%	22.6%	0.3%	2.6%
(32)	*Newswire*	22.5%	25.0%	0.3%	2.5%
(33)	*Newswire*	25.0%	27.4%	0.3%	2.4%

Table S4. **Performance validation of our model** across Admin, Engineer, Finance, Marketing (all predating ChatGPT's launch), using a blend of official human and LLM-assisted job postings.

No.	Validation Data Category	Ground Truth α	Estimated		Prediction Error
			α	CI ($\pm$)	
(1)	*Admin*	0.0%	1.2%	0.5%	1.2%
(2)	*Admin*	2.5%	4.0%	0.6%	1.5%
(3)	*Admin*	5.0%	6.6%	0.7%	1.6%
(4)	*Admin*	7.5%	9.1%	0.7%	1.6%
(5)	*Admin*	10.0%	11.6%	0.8%	1.6%
(6)	*Admin*	12.5%	14.1%	0.8%	1.6%
(7)	*Admin*	15.0%	16.7%	0.8%	1.7%
(8)	*Admin*	17.5%	19.1%	0.8%	1.6%
(9)	*Admin*	20.0%	21.6%	0.9%	1.6%
(10)	*Admin*	22.5%	24.0%	0.9%	1.5%
(11)	*Admin*	25.0%	26.4%	0.9%	1.4%
(12)	*Engineer*	0.0%	0.9%	0.5%	0.9%
(13)	*Engineer*	2.5%	3.6%	0.6%	1.1%
(14)	*Engineer*	5.0%	6.2%	0.7%	1.2%
(15)	*Engineer*	7.5%	8.8%	0.8%	1.3%
(16)	*Engineer*	10.0%	11.3%	0.8%	1.3%
(17)	*Engineer*	12.5%	13.8%	0.8%	1.3%
(18)	*Engineer*	15.0%	16.4%	0.9%	1.4%
(19)	*Engineer*	17.5%	18.9%	0.8%	1.4%
(20)	*Engineer*	20.0%	21.4%	0.9%	1.4%
(21)	*Engineer*	22.5%	23.9%	0.9%	1.4%
(22)	*Engineer*	25.0%	26.4%	0.9%	1.4%
(23)	*Finance*	0.0%	0.7%	0.4%	0.7%
(24)	*Finance*	2.5%	3.5%	0.6%	1.0%
(25)	*Finance*	5.0%	6.0%	0.7%	1.0%
(26)	*Finance*	7.5%	8.5%	0.7%	1.0%
(27)	*Finance*	10.0%	10.9%	0.7%	0.9%
(28)	*Finance*	12.5%	13.4%	0.7%	0.9%
(29)	*Finance*	15.0%	15.9%	0.8%	0.9%
(30)	*Finance*	17.5%	18.3%	0.8%	0.8%
(31)	*Finance*	20.0%	20.7%	0.9%	0.7%
(32)	*Finance*	22.5%	23.1%	0.8%	0.6%
(33)	*Finance*	25.0%	25.5%	0.9%	0.5%
(23)	*Marketing*	0.0%	0.6%	0.5%	0.6%
(24)	*Marketing*	2.5%	3.4%	0.6%	0.9%
(25)	*Marketing*	5.0%	5.9%	0.6%	0.9%
(26)	*Marketing*	7.5%	8.4%	0.7%	0.9%
(27)	*Marketing*	10.0%	10.9%	0.8%	0.9%
(28)	*Marketing*	12.5%	13.4%	0.8%	0.9%
(29)	*Marketing*	15.0%	15.8%	0.8%	0.8%
(30)	*Marketing*	17.5%	18.3%	0.9%	0.8%
(31)	*Marketing*	20.0%	20.8%	0.8%	0.8%
(32)	*Marketing*	22.5%	23.3%	0.9%	0.8%
(33)	*Marketing*	25.0%	25.7%	0.9%	0.7%

Table S5. **Performance validation of our model** across Operation, Sales, Scientist (all predating ChatGPT's launch), using a blend of official human and LLM-assisted job postings.

No.	Validation Data Category	Ground Truth α	Estimated		Prediction Error
			α	CI ($\pm$)	
(1)	*Operation*	0.0%	0.8%	0.5%	0.8%
(2)	*Operation*	2.5%	3.3%	0.6%	0.8%
(3)	*Operation*	5.0%	5.9%	0.7%	0.9%
(4)	*Operation*	7.5%	8.4%	0.7%	0.9%
(5)	*Operation*	10.0%	10.9%	0.8%	0.9%
(6)	*Operation*	12.5%	13.3%	0.8%	0.8%
(7)	*Operation*	15.0%	15.8%	0.8%	0.8%
(8)	*Operation*	17.5%	18.2%	0.9%	0.7%
(9)	*Operation*	20.0%	20.7%	0.9%	0.7%
(10)	*Operation*	22.5%	23.2%	0.9%	0.7%
(11)	*Operation*	25.0%	25.6%	0.9%	0.6%
(12)	*Sales*	0.0%	1.2%	0.5%	1.2%
(13)	*Sales*	2.5%	3.7%	0.6%	1.2%
(14)	*Sales*	5.0%	6.2%	0.7%	1.2%
(15)	*Sales*	7.5%	8.6%	0.8%	1.1%
(16)	*Sales*	10.0%	11.0%	0.8%	1.0%
(17)	*Sales*	12.5%	13.4%	0.8%	0.9%
(18)	*Sales*	15.0%	15.8%	0.8%	0.8%
(19)	*Sales*	17.5%	18.2%	0.8%	0.7%
(20)	*Sales*	20.0%	20.7%	0.9%	0.7%
(21)	*Sales*	22.5%	23.1%	0.9%	0.6%
(22)	*Sales*	25.0%	25.5%	0.9%	0.5%
(23)	*Scientist*	0.0%	2.0%	0.6%	2.0%
(24)	*Scientist*	2.5%	4.8%	0.7%	2.3%
(25)	*Scientist*	5.0%	7.3%	0.7%	2.3%
(26)	*Scientist*	7.5%	9.8%	0.8%	2.3%
(27)	*Scientist*	10.0%	12.3%	0.8%	2.3%
(28)	*Scientist*	12.5%	14.7%	0.9%	2.2%
(29)	*Scientist*	15.0%	17.2%	0.9%	2.2%
(30)	*Scientist*	17.5%	19.7%	1.0%	2.2%
(31)	*Scientist*	20.0%	22.1%	0.9%	2.1%
(32)	*Scientist*	22.5%	24.5%	1.0%	2.0%
(33)	*Scientist*	25.0%	27.0%	1.0%	2.0%
